# Exploring the Diversity of Field Strains of *Brucella abortus* Biovar 3 Isolated in West Africa

**DOI:** 10.3389/fmicb.2017.01232

**Published:** 2017-06-30

**Authors:** Moussa Sanogo, David Fretin, Eric Thys, Claude Saegerman

**Affiliations:** ^1^Central Veterinary Laboratory of Bingerville, LANADABingerville, Ivory Coast; ^2^Department of Bacteriology and Immunology, Veterinary and Agro-chemical Research CentreBrussels, Belgium; ^3^Department of Biomedical Sciences, Institute of Tropical MedicineAntwerp, Belgium; ^4^Research Unit of Epidemiology and Risk Analysis Applied to Veterinary Sciences (UREAR-ULg), Faculty of Veterinary Medicine, Fundamental and Applied Research for Animal and Health Center, University of LiègeLiège, Belgium

**Keywords:** molecular epidemiology, brucellosis, *Brucella*, *Brucella abortus* biovar 3, biotyping, MLVA, West Africa

## Abstract

Brucellosis is one of the most widespread bacterial zoonotic diseases in the world, affecting both humans and domestic and wild animals. Identification and biotyping of field strains of *Brucella* are of key importance for a better knowledge of the epidemiology of brucellosis, for identifying appropriate antigens, for managing disease outbreaks and for setting up efficient preventive and control programmes. Such data are required both at national and regional level to assess potential threats for public health. Highly discriminative genotyping methods such as the multiple locus variable number of tandem repeats analysis (MLVA) allow the comparison and assessment of genetic relatedness between field strains of *Brucella* within the same geographical area. In this study, MLVA biotyping data retrieved from the literature using a systematic review were compared using a clustering analysis and the Hunter-Gaston diversity index (HGDI). Thus, the analysis of the 42 MLVA genotyping results found in the literature on West Africa [i.e., from Ivory Coast (1), Niger (1), Nigeria (34), The Gambia (3), and Togo (3)] did not allow a complete assessment of the actual diversity among field strains of *Brucella*. However, it provided some preliminary indications on the co-existence of 25 distinct genotypes of *Brucella abortus* biovar 3 in this region with 19 genotypes from Nigeria, three from Togo and one from Ivory Coast, The Gambia, and Niger. The strong and urgent need for more sustainable molecular data on prevailing strains of *Brucella* in this sub-region of Africa and also on all susceptible species including humans is therefore highlighted. This remains a necessary stage to allow a comprehensive understanding of the relatedness between field strains of *Brucella* and the epidemiology of brucellosis within West Africa countries.

## Introduction

Brucellosis is one of the most widespread bacterial zoonotic diseases in the world, affecting both humans and domestic and wild animals (Maurin, 2005; Corbel, 2006). The disease is caused by Gram-negative facultative intracellular bacteria of the genus *Brucella*. According to the World Health Organization (WHO), about 500,000 new cases of human brucellosis are reported annually worldwide (Corbel, 1997; Pappas et al., 2006). In animals, brucellosis is responsible for many economic losses because of abortions, decrease in production (particularly reduced milk production), losses of calves, viable but weak calves, reproductive disorders, and costs of intervention. With its impact on productivity, this disease contributes worsening the deficit of animal protein especially for populations in developing countries, where food needs are continuously increasing (Perry, 2002). In areas where people's livelihood heavily depends on livestock, the impact of brucellosis might therefore exacerbate poverty (Cáceres, 2010).

In spite of its status as a neglected tropical disease, bovine brucellosis remains the most widespread disease in animals and the main concern in Sub-Saharan African countries (Akakpo and Bornarel, 1987; Corbel, 1997; McDermott and Arimi, 2002; Bronsvoort et al., 2009). For a better understanding of the epidemiology of bovine brucellosis, phenotypic, and genotypic knowledge on prevailing *Brucella* spp. are required in both human and animal hosts. Thus, *Brucella* causing brucellosis has been investigated throughout the years in different regions of the world including West Africa. In this part of Africa, the presence and the endemicity of brucellosis were confirmed, with *Brucella abortus* biovar 3 being the most commonly isolated strains in cattle (Sanogo et al., 2013a).

This paper compares and investigates the relatedness between the prevailing field strains of *B. abortus* biovar 3 from West Africa.

## Materials and methods

### Study area

**Figure 1:**
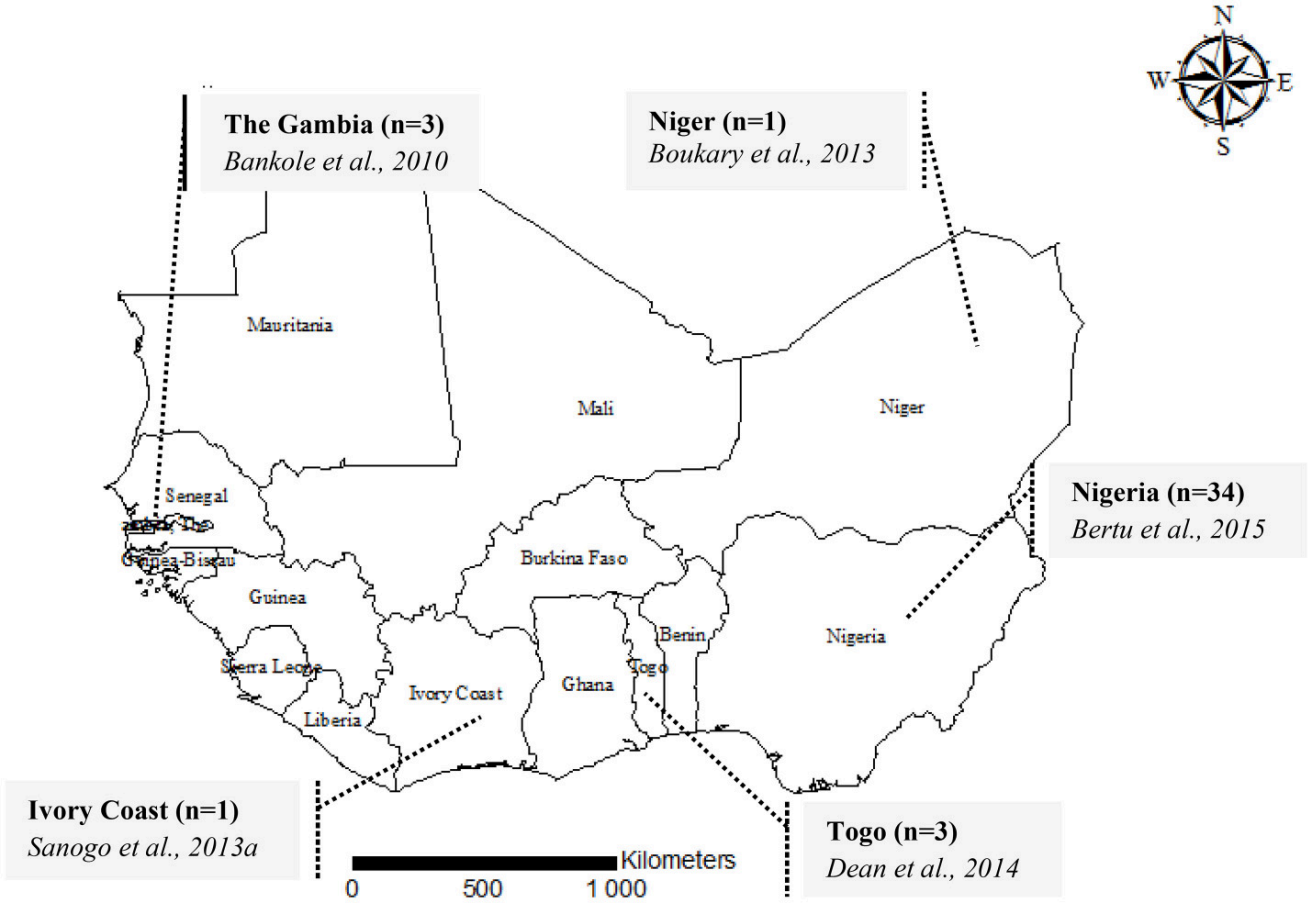
A map showing the geographical origin of MLVA genotyping data of *Brucella abortus biovar 3* in West Africa, 2015. For each country, the number of isolates genotyped [i.e., Ivory Coast (*n* = 1)] is provided followed by the authors and the year of publication (i.e., Sanogo et al., 2013b).

With a surface area of 5 112 903 km^2^ representing a fifth of the African continent, West Africa is one of the four major regions of Sub-Saharan Africa. This region includes 14 countries including Benin, Burkina Faso, Cape Verde, The Gambia, Ghana, Guinea, Ivory Coast, Liberia, Mali, Niger, Nigeria, Senegal, Sierra Leone, and Togo (Figure 1). These countries comprise almost 25% of the cattle population of the continent with about 70 million heads of cattle of different types (*Bos taurus* type, *Bos indicus* type and crossbreds) (FAO, 2017). These cattle are mostly raised extensively in sedentary herds. This region is also characterized by the existence of frequent livestock movements between countries through transhumance or commercial exchanges.

### Prevailing field strains of *Brucella abortus* biovar 3 from West Africa

**Figure 2:**
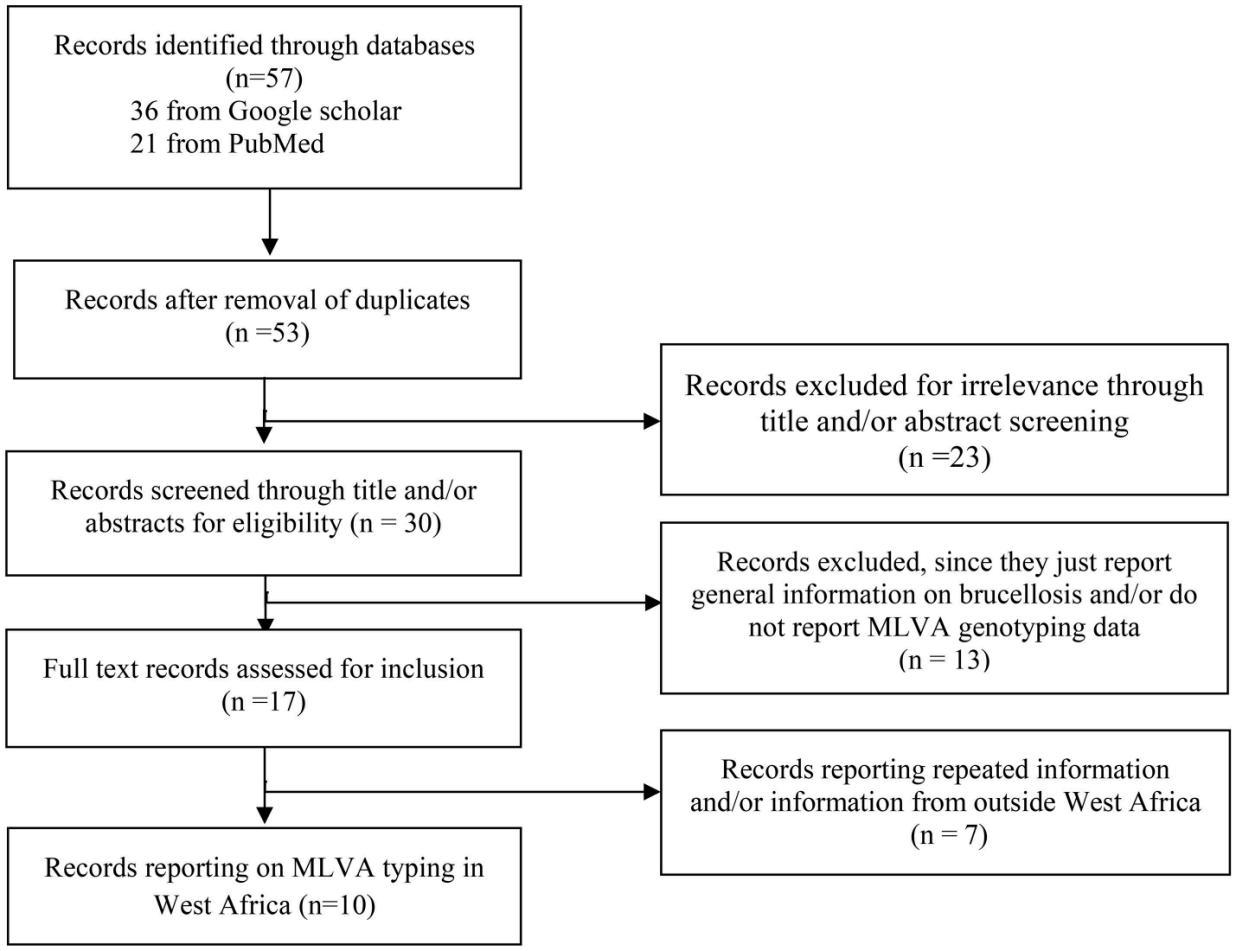
Flow diagram presenting a summary of the literature search on genotyping of field strains of *Brucella* from West Africa and other parts of Africa.

A Preferred Reporting Items of Systematic reviews and Meta-Analyses (PRISMA) approach (Moher et al., 2009) was used to identify available and accessible information in the literature on typing of prevailing field strains of *Brucella* in both human and animals through general internet search engines, including Google Scholar and PubMed, with no language and time period restrictions. The search strategy was adapted according to the database. Search terms were composed by combinations of keywords. In Google Scholar, “Brucellosis+*Brucella*+MLVA+typing+genotyping+Sub-saharan+Africa” was used while in PubMed, the following search algorithm was used: ((((Brucellosis) OR *Brucella*)) AND (((genotyping) OR typing) OR MLVA)) AND ((Africa) OR sub-Saharan Africa). Firstly, titles and abstracts were screened and available full texts were screened for relevant information. Thus, studies reporting information on genotyping data of field strains of *B. abortus* biovar 3 from sub-Saharan Africa and especially West Africa were considered and were given a particular focus for final inclusion. When provided, Multiple Locus Variable number of tandem repeats Analysis (MLVA) data [e.g., the number of repeats in a set of variable number of tandem repeats (VNTR) loci] were extracted from the selected paper, summarized, and subjected to further analysis. A flow diagram summarizing the literature search strategy is presented in Figure 2.

### Multiple locus variable number of tandem repeats analysis

MLVA profiles of field strains of *B. abortus* biovar 3 isolated from West Africa were used in this study (Figure 1). Briefly, MLVA consists of the assessment of the number of repeats in a set of variable number of tandem repeats (VNTR) loci. In MLVA 16, two sets of VNTRs gathered into 8 microsatellite markers (panel 1: Bruce06, Bruce08, Bruce11, Bruce12, Bruce42, Bruce43, Bruce45, Bruce55) and 8 microsatellite markers (panel 2) comprising two groups (panel 2A: Bruce18, Bruce19, Bruce21; and panel 2B: Bruce04, Bruce07, Bruce09, Bruce16, Bruce30) are examined (Le Flêche et al., 2006; Maquart et al., 2009). The number of repetitions of each locus of each panel, constituting the MLVA profile, is derived from the size of the band of the PCR products (Le Flêche et al., 2006).

### Comparison of MLVA profiles

Diversity and relatedness among field strains of *B. abortus* biovar 3 from West Africa were assessed by calculating the Hunter-Gaston diversity index (HGDI), a numerical index measuring the probability that two strains consecutively taken from a given population would be placed into different typing groups (Hunter and Gaston, 1988) (http://www.hpa-bioinformatics.org.uk/cgi-bin/DICI/DICI.pl). The relatedness between the distinct MLVA profiles of West African strains and neighbor profiles originating from Africa in the public MLVA *Brucella* database on MLVAnet (http://mlva.u-psud.fr/brucella/) was also assessed with a Ward hierarchical clustering analysis using the *hclust* function and the *cluster* package in R software (http://www.r-project.org). Using results of a Ward linkage clustering analysis of the number of variable tandem repeats, a dendrogram of clustered MLVA profiles of West African strains was also generated. In order to assess potential relatedness with others prevailing strains from sub-Saharan Africa, comparison of the 25 distinct MLVA profiles from West Africa includes three lately published *B. abortus* biovar 3 MLVA profiles from Tanzania (Mathew et al., 2015) and five other sub-Saharan Africa *B. abortus* biovar 3 field strains and neighbor profiles from the *Brucella* MLVA database, namely Kenya (Muendo et al., 2012), Sudan, Uganda, and Chad (Le Flêche et al., 2006) (Table 1).

**Table 1:** Multiple Loci Variable Number Tandem Repeats analysis (MLVA) distinct profiles of West African isolates of *B. abortus* biovar 3 and some close neighbor profiles from Africa retrieved from literature and from the *Brucella* MLVA bank.

**id**	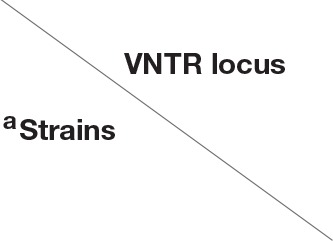	**Panel 1**								**Panel 2A**			**Panel 2B**					**Period of collection**	**Location**	**Host**	**References**
		**Bruce06**	**Bruce08**	**Bruce11**	**Bruce12**	**Bruce42**	**Bruce43**	**Bruce45**	**Bruce55**	**Bruce18**	**Bruce19^b^**	**Bruce21**	**Bruce04**	**Bruce07**	**Bruce09**	**Bruce16**	**Bruce30**				
1	Ref Strain Tulya	3	5	5	11	2	2	3	3	8	40	8	6	5	3	11	5	1958	Uganda	Human	Le Flêche et al., 2006
2	^c^Ref Strain BCCN 93_26	3	5	5	11	2	2	3	3	6	40	8	6	8	3	7	7	1993	Sudan	Dromedary	Le Flêche et al., 2006
3	^d^Ref Strain BfR7	3	5	5	11	2	2	3	3	6	–	8	6	4	3	8	4	–	Chad	Cattle	Le Flêche et al., 2006
4	^e^IVC_isolate	3	5	4	11	2	2	3	3	7	21	8	4	5	3	7	3	2009	Ivory Coast	Cattle	Sanogo et al., 2013b
5	Niger_isolate	3	5	3	11	2	2	3	3	8	21	8	6	2	3	12	7	2009	Niger	Cattle	Boukary et al., 2013
6	The_gambia_isolate	3	5	4	11	2	2	3	3	7	–	8	5	5	3	3	5	–	The Gambia	Cattle	Bankole et al., 2010
7	Togo_1	3	5	3	11	2	2	3	3	10	41	8	4	2	3	8	4	2011–2012	Togo	Cattle	Dean et al., 2014
8	Togo_2	3	5	3	11	2	2	3	3	8	41	8	4	2	3	5	4	2011–2012	Togo	Cattle	Dean et al., 2014
9	Togo_3	3	5	3	11	2	2	3	3	8	41	8	4	2	3	6	4	2011–2012	Togo	Cattle	Dean et al., 2014
10	Nigeria_602	3	4	4	12	2	1	3	3	6	40	8	7	4	3	4	5	1976–2012	Nigeria	Cattle	Bertu et al., 2015
11	Nigeria_603	3	4	4	11	2	1	3	3	6	40	8	7	4	3	4	6	1976–2012	Nigeria	Cattle	Bertu et al., 2015
12	Nigeria_604	3	4	4	11	2	2	3	3	6	40	9	8	4	3	3	5	1976–2012	Nigeria	Cattle	Bertu et al., 2015
13	Nigeria_605	3	4	4	11	2	2	3	3	6	42	8	7	6	3	6	5	1976–2012	Nigeria	Cattle	Bertu et al., 2015
14	Nigeria_606	3	4	4	11	2	2	3	3	6	40	8	8	4	3	5	6	1976–2012	Nigeria	Cattle	Bertu et al., 2015
15	Nigeria_607	3	4	4	11	2	2	3	3	6	40	8	8	4	4	7	5	1976–2012	Nigeria	Sheep	Bertu et al., 2015
16	Nigeria_608	3	4	4	10	2	2	3	3	6	40	8	8	4	4	8	5	1976–2012	Nigeria	Cattle	Bertu et al., 2015
17	Nigeria_609	3	4	4	10	2	3	3	3	6	40	8	6	7	4	8	8	1976–2012	Nigeria	Cattle	Bertu et al., 2015
18	Nigeria_610	3	4	4	10	2	2	3	3	6	40	8	5	10	3	14	6	1976–2012	Nigeria	Cattle	Bertu et al., 2015
19	Nigeria_611	3	4	4	10	2	2	3	3	6	40	8	5	10	3	9	6	1976–2012	Nigeria	Cattle	Bertu et al., 2015
20	Nigeria_612	3	4	4	11	2	2	3	3	6	42	9	8	3	3	8	5	1976–2012	Nigeria	Sheep	Bertu et al., 2015
21	Nigeria_613	4	4	4	12	2	2	3	3	6	44	9	5	10	3	8	6	1976–2012	Nigeria	Cattle	Bertu et al., 2015
22	Nigeria_614	3	3	4	12	2	1	3	3	7	46	9	5	9	3	8	7	1976–2012	Nigeria	Cattle	Bertu et al., 2015
23	Nigeria_615	3	3	4	12	2	1	3	3	7	46	9	8	2	3	6	5	1976–2012	Nigeria	Cattle	Bertu et al., 2015
24	Nigeria_616	3	3	4	12	2	1	3	3	7	48	9	7	3	1	6	5	1976–2012	Nigeria	Cattle	Bertu et al., 2015
25	Nigeria_617	3	5	4	11	2	1	3	3	7	40	8	5	10	3	4	6	1976–2012	Nigeria	Cattle	Bertu et al., 2015
26	Nigeria_618	3	5	4	11	2	2	3	3	6	40	8	8	4	3	4	5	1976–2012	Nigeria	Horse	Bertu et al., 2015
27	Nigeria_619	3	5	4	11	2	1	3	3	6	40	8	5	10	3	4	6	1976–2012	Nigeria	Cattle	Bertu et al., 2015
28	Nigeria_620	4	5	4	12	2	2	3	3	7	40	8	8	4	3	4	5	1976–2012	Nigeria	Horse	Bertu et al., 2015
29	Kenya_11-KEBa2	3	5	4	11	2	2	3	3	7	40	8	6	5	3	12	5	2009	Kenya	Cattle	Muendo et al., 2012
30	Kenya_12-KEBa1	3	5	4	11	2	2	3	3	7	40	8	6	6	3	11	6	2009	Kenya	Cattle	Muendo et al., 2012
31	Tanzania_C64	2	4	2	12	3	2	3	3	5	–	8	7–8	2	6	7	4	2012–2013	Tanzania	Cattle	Mathew et al., 2015
32	Tanzania_C65	2	4	2	12	3	2	3	3	5	42–44	8	7–8	2	6	8	4	2012–2013	Tanzania	Cattle	Mathew et al., 2015
33	Tanzania_C66	2	4	2	12	3	2	3	3	5	42–44	8	7–8	2	6	8	4	2012–2013	Tanzania	Cattle	Mathew et al., 2015

^a^For each country, when the same MLVA profile is shared by more than one strain, only one genotype or distinct MLVA profile is presented. Here the 25 distinct genotypes of Brucella abortus biovar 3 obtained from 42 MLVA profiles found in the literature on West Africa (19 genotypes out of 34 profiles from Nigeria, 3 out 3 profiles from Togo, 1 distinct genotype out of 3 profiles from the Gambia, 2 from each single profile reported from Ivory Coast and Niger) are presented with 5 strains from East Africa and 3 references strains from the MLVA bank;

b additional locus comprised in the MLVA-16 and absent in MLVA-15;

c Brucella Culture Collection;

d Federal Institute for Risk Assessment;

e *Isolate from Ivory Coast*.

## Results

In order to explore the genetic diversity of field strains of *B. abortus* biovar 3 from West Africa, available and accessible MLVA genotyping data were retrieved from the literature (Figures 1, 2). Among 57 published papers initially retrieved from the literature search, only 10 papers report MLVA genotyping data of West African *B abortus* biovar 3 strains. These 10 papers include four review papers reporting already published data covering sub-Saharan Africa in general (Boukary et al., 2013; Ducrotoy et al., 2017) and West Africa in particular (Sanogo et al., 2013a; Dean et al., 2014). Except from Nigeria, where strains came from both imported and autochthonous cattle and from sheep (*n* = 2) and horse (*n* = 2), strains originating from other countries were obtained from autochthonous cattle. None of the retrieved MLVA profiles were reported from humans so far. A total of 42 MLVA genotyping results were reported in the literature. Comparison of MLVA profiles of *B. abortus* biovar 3 field strains reported so far within West Africa revealed the presence of 25 distinct genotypes [e.g., a single genotype from the three strains isolated from The Gambia (Bankole et al., 2010), one from the unique strain from Niger (Boukary et al., 2013), one from the unique strain from Ivory Coast (Sanogo et al., 2013b), three genotypes from the three strains from Togo (Dean et al., 2014), and 19 genotypes from the 34 strains from Nigeria (Bertu et al., 2015)] (Figure 1).

While considering only panel 1 (MLVA 8), which is indicative of the species, diversity indexes of 0.620 (95% CI: 0.532–0.708), 0.580 (95% CI: 0.428–0.732), 0.477 (95% CI: 0.316–0.638), 0.280 (95% CI: 0.085–0.475), and 0.153 (95% CI: 0.000–0.332) were observed, respectively, at locus Bruce08, Bruce12, Bruce43, Bruc11, and Bruc06 with different genotypes. The others loci showed identical number of repeating units among the genotypes observed (e.g., Bruce42: 1; Bruce45: 1; Bruce55: 1) (Table 2). Highest diversity indexes were observed with the set of markers composing panel 2, especially at Bruce16 (HGDI = 0.870, 95% CI: 0.808–0.932), known as one of the most variable locus. Within this panel 2, while considering highly discriminative markers (i.e., Bruce04, Bruce07, Bruce09, Bruce16, and Bruce30), three to nine different alleles were found.

**Table 2:** The Hunter Gaston Diversity Index for different loci of West African field strains of *B. abortus* biovar 3 (i.e., from Ivory Coast, Niger, Nigeria, The Gambia, and Togo) based on MLVA 16 data.

**Panel**		**Locus**	**Diversity index**	**95% Confidence interval**	**Number of alleles**	**Max(pi)**
Panel 1		Bruce06	0.153	0.000–0.332	2	0.920
		Bruce08	0.620	0.532–0.708	3	0.480
		Bruce11	0.280	0.085–0.475	2	0.840
		Bruce12	0.580	0.428–0.732	3	0.600
		Bruce42	0.000	0.000–0.237	1	1.000
		Bruce43	0.477	0.316–0.638	3	0.680
		Bruce45	0.000	0.000–0.237	1	1.000
		Bruce55	0.000	0.000–0.237	1	1.000
Panel 2	Panel 2a	Bruce18	0.617	0.474–0.759	4	0.560
		Bruce19	0.720	0.547–0.893	8	0.520
		Bruce21	0.380	0.206–0.554	2	0.760
	Panel 2b	Bruce04	0.793	0.730–0.857	5	0.320
		Bruce07	0.833	0.755–0.912	8	0.320
		Bruce09	0.290	0.077–0.503	3	0.840
		Bruce16	0.870	0.808–0.932	9	0.240
		Bruce30	0.733	0.617–0.849	6	0.440

*Diversity Index (for VNTR data): a measure of the variation of the number of repeats at each locus. It ranges from 0.0 (no diversity) to 1.0 (complete diversity)*.

*Confidence Interval: precision of the Diversity Index, expressed as 95% upper and lower boundaries*.

*max (pi): fraction of samples that have the most frequent repeat number in this locus (range 0.0–1.0)*.

## Discussion

For many years, *Brucella* spp. causing bovine brucellosis were characterized using both phenotypic and genotypic methods. While *B. abortus* biovar 1 have been reported as the most encountered in cattle worldwide (Corbel, 1997), in the USA (Bricker et al., 2003), and in Latin America (Acha and Szyfres, 2003; Lucero et al., 2008; Minharro et al., 2013), *B. abortus* biovar 3 was predominant in both native cattle and buffalo from eastern Africa and China (Timm, 1982; Domenech et al., 1983). *B. abortus* biovar 3 was also identified as the most commonly isolated in cattle from West Africa and Sub-Saharan Africa (Sanogo et al., 2013a; Bertu et al., 2015). In West Africa, where only *B. abortus* was reported so far, field strains of *B. abortus* biovar 3 were characterized mostly in cattle using a combination of bacteriological phenotypic typing and MLVA genotyping approaches. These West African isolates were mostly characterized from autochthonous cattle and from hygroma fluid samples. Phenotypic methods consisted of bacteriological isolation and identification and relied on a combination of morphological, cultural, serological and biochemical characteristics in order to characterize suspicious colonies (Alton et al., 1988). However, phenotypic typing methods may fail to correctly classify or differentiate some strains as in Nigeria (Bertu et al., 2015). Therefore, conventional bacteriological identification needs to be supplemented by molecular methods such as the VNTR analysis (MLVA). MLVA is a powerful molecular tool for typing and for assessing the potential relationships between *Brucella* spp. isolates from different sources of infection and from different geographical origins. It is a particularly useful method to study the molecular epidemiology of *Brucella* where a high discriminatory power is required (Bricker et al., 2003; Cutler et al., 2005; Le Flêche et al., 2006). Wherever possible, more accurate and discriminative typing methods such as the enhanced AMOS-ery PCR and MLVA should be used in complementarity with conventional biotyping methods (Ocampo-Sosa et al., 2005; Bankole et al., 2010; Sanogo et al., 2013b; Dean et al., 2014; Bertu et al., 2015).

**Figure 3:**
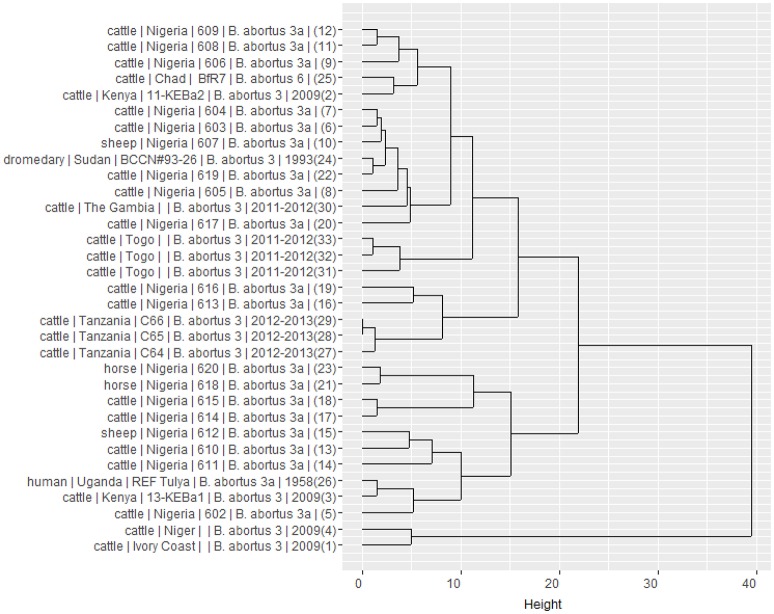
Dendrogram clustered MLVA profiles showing the relation between the 25 West African isolates of *B. abortus* biovar 3 and eight neighbor profiles from Africa retrieved in the literature and from the *Brucella* MLVA bank. It is built from results of a Ward linkage cluster analysis of the number of variable tandem repeats (VNTR) of the MLVA 16 loci. For each strain, information on host, country of origin, strain reference, species and biovar, year of isolation (when available) and number of order in the database are provided.

Using panel 1 (MLVA8), 10 genotypes were obtained while 18 genotypes were obtained using the combination of panel 1 and 2B (MLVA11). The analysis of the complete MLVA16 (panels 1, 2A and 2B) revealed 25 distinct genotypes. Clustering analysis of the different MLVA profiles suggested the co-existence of distinct clonal complexes (Figure 3). While the three strains isolated from The Gambia shared the same profile, distinct profiles co-existed in Nigeria and Togo. The Togolese strains appeared to be related to many Nigerian strains and isolates from The Gambia. On the other hand, isolates from Niger and Ivory Coast appeared to be genetically related. In Nigeria where distinct profiles also co-exist, some isolates were more related to eastern African isolates originating from Tanzania and Kenya. These observations might suggest a possible relation between African *B. abortus* biovar 3 strains. Indeed, despite the relative limited number of strains compared, these results provide some preliminary indications on the co-existence of different genetic profiles among the prevailing field strains of *B. abortus* biovar 3 in this sub-region (Dean et al., 2014; Bertu et al., 2015). This heterogeneity among *B. abortus* biovar 3 strains originating from Africa was already described, with the North African strains more closely related to European *B. abortus* biovar 3b strain lineage and the sub-Saharan African strains more related to *B. abortus* biovar 3a lineage (Ocampo-Sosa et al., 2005; Ica et al., 2008; Bertu et al., 2015; Mathew et al., 2015; Ducrotoy et al., 2017). However, despite the genotypic diversity observed, the closeness of most of sub-Saharan African strains with the human reference Tulya strain from Uganda, put forward the hypothesis of the possible dominance of lineage 3a among West African *B. abortus* biovar 3 (Bertu et al., 2015; Ducrotoy et al., 2017) and a possible common historical origin of brucellosis in this region. Indeed, this lineage commonly isolated in West Africa is known to be confined in the African continent where *B. abortus* is believed to originate (Whatmore et al., 2016). Such a hypothesis associated with the observed polymorphisms is in line with unrestricted livestock movements through transhumance and trade among countries composing this sub-region (OECD, 2008), which might favor frequent introduction and reintroduction of the pathogen. So far, data on prevailing strains of *Brucella* in both animal and human hosts are still scare and irregularly reported (Sanogo et al., 2013a). In order to challenge such hypothesis and allow a better understanding of the epidemiology of brucellosis in West Africa, more molecular typing results are needed. In West Africa, brucellosis (or evidence of its presence) was reported in most of the 14 countries so far (Mangen et al., 2002; Boukary et al., 2013). Adequate and efficient control of brucellosis in this region implies a comprehensive understanding of its epidemiology at West African region scale, in order to include prevailing strains causing the disease and adjust diagnostic tools. Indeed, additional data on prevailing field strain of *Brucella* are required to identify the sources of infection and to understand the transmission pathways of this infection between animals and from animal to humans (Adone and Pasquali, 2013).

In conclusion, the number of strains analyzed in this study precludes an actual complete and comprehensive assessment of the relatedness of field strains of *B. abortus* biovar 3 in cattle from West Africa but provides preliminary indications on the co-existence of distinct profiles in this sub-region, in line with other recent findings (Bertu et al., 2015; Ducrotoy et al., 2017). More extended knowledge of prevailing strains in livestock and other hosts remains necessary to actually assess their diversity and to fully understand the molecular epidemiology of *Brucella* infection, distribution, and transmission patterns within West Africa and across the whole African continent (Godfroid et al., 2013). By allowing comparison among strains, MLVA genotyping methods would also be useful as a surveillance tool of the distribution of brucellosis in West Africa, where frequent movements of livestock between countries are expected to play a role in the spread of *Brucellae*. So far, MLVA data from West Africa are not available from MLVA public database. It might be therefore suggested that studies publishing MLVA typing results explicitly report, share details profiles and be more informative. This is particularly critical for a sub-region where resources and molecular epidemiological investigations are limited.

Formal collaboration between countries and their respective public health actors is required for sharing available information and for implementing harmonized surveillance and control strategies. Such collaboration coupled with adoption of the concept of “*One health approach*” would be particularly beneficial in a regional framework, especially in West Africa where national resources and capabilities for prevention, control and surveillance of infectious diseases of public importance such as brucellosis are still scarse (Saegerman et al., 2010, 2012; Marcotty et al., 2013). It is also essential to ensure a sustainable system of data collection on prevailing strains covering the whole West African region with a better coverage of other susceptible domestic and wild animals in order to document sources of human infections and to produce strong molecular evidence informing on the epidemiologic links between strains of *Brucella* within this region.

## Author contributions

MS performed the literature review and the clustering analyses. MS and CS wrote the manuscript and all authors including DF and ET reviewed, commented and approved the final manuscript.

### Conflict of interest statement

The authors declare that the research was conducted in the absence of any commercial or financial relationships that could be construed as a potential conflict of interest.
